# Supplementary material for: The Microbial Composition of Bovine Colostrum as Influenced by Antibiotic Treatment
Source: Antibiotics (Basel). 2025 Dec 3;14(12):1217. doi: 10.3390/antibiotics14121217 (PMC12729519; doi:10.3390/antibiotics14121217)
Supplement: Supplementary file 1 [file antibiotics-14-01217-s001.zip › antibiotics-3974888-supplementary.pdf]

Supplementary Table S1: Metadata on the colostrum samples. All cows were housed indoors during the winter period until calving (November – February). The breed of cows was the same on all farms (Holstein Friesian).

Sample ID	Farm	Parity Status	Age (years)	Antibiotic Treatment
**C10**	Farm C	2	4	None
**C11**	Farm C	4+	6	None
**C12**	Farm C	1	3	None
**C13**	Farm C	1	3	None
**C14**	Farm C	4+	7	None
**C15**	Farm C	2	4	None
**C16**	Farm C	0	2	None
**C17**	Farm C	4+	8	None
**C18**	Farm C	4+	6	None
**C19**	Farm C	0	2	None
**C1**	Farm C	1	3	None
**C20**	Farm C	4+	6	None
**C2**	Farm C	0	2	None
**C3**	Farm C	1	3	None
**C5**	Farm C	0	2	None
**C6**	Farm C	3	5	None
**C7**	Farm C	1	3	None
**C8**	Farm C	4+	6	None
**C9**	Farm C	4+	6	None
**D10**	Farm D	4+	10	Cefquinome
**D11**	Farm D	2	4	Cefquinome
**D12**	Farm D	4+	6	Cefquinome
**D13**	Farm D	1	3	Cefquinome
**D14**	Farm D	1	3	Cefquinome
**D15**	Farm D	3	5	Cefquinome
**D17**	Farm D	2	4	Cefquinome
**D18**	Farm D	4+	9	Cefquinome
**D19**	Farm D	4+	6	Cefquinome
**D1**	Farm D	4+	10	Cefquinome
**D20**	Farm D	0	2	None
**D2**	Farm D	3	5	Cefquinome
**D3**	Farm D	2	4	Cefquinome
**D5**	Farm D	4+	9	Cefquinome
**D6**	Farm D	4+	6	Cefquinome
**D7**	Farm D	3	5	Cefquinome
**D9**	Farm D	1	3	Cefquinome
**L10**	Farm L	3	5	Ubro Red
**L11**	Farm L	3	5	Ubro Red

**L12**	Farm L	4+	7	Ubro Red
**L13**	Farm L	1	3	Ubro Red
**L14**	Farm L	4+	8	Ubro Red
**L15**	Farm L	0	2	None
**L16**	Farm L	3	5	Ubro Red
**L18**	Farm L	4+	9	Ubro Red
**L19**	Farm L	2	4	Ubro Red
**L20**	Farm L	2	4	Ubro Red
**L2**	Farm L	3	5	Ubro Red
**L3**	Farm L	1	3	Ubro Red
**L5**	Farm L	1	3	Ubro Red
**L6**	Farm L	1	3	Ubro Red
**L7**	Farm L	2	4	Ubro Red
**L8**	Farm L	4+	7	Ubro Red
**L9**	Farm L	4+	9	Ubro Red
**P10**	Farm P	1	3	Cefquinome
**P11**	Farm P	4+	8	Cefquinome
**P12**	Farm P	2	4	Cefquinome
**P13**	Farm P	4+	7	Cefquinome
**P14**	Farm P	0	2	None
**P15**	Farm P	4+	9	Ubro Red
**P16**	Farm P	0	2	None
**P18**	Farm P	0	2	None
**P19**	Farm P	1	3	Cefquinome
**P1**	Farm P	4+	6	Cefquinome
**P20**	Farm P	1	3	Cefquinome
**P2**	Farm P	2	4	Cefquinome
**P3**	Farm P	4+	6	Cefquinome
**P4**	Farm P	4+	8	Cefquinome
**P5**	Farm P	4+	7	Cefquinome
**P6**	Farm P	4+	7	Cefquinome
**P7**	Farm P	4+	6	Cefquinome
**P8**	Farm P	2	4	Cefquinome
**P9**	Farm P	1	3	Cefquinome
**T10**	Farm T	3	5	Ubro Red
**T11**	Farm T	0	2	None
**T12**	Farm T	4+	13	Ubro Red
**T13**	Farm T	4+	9	Ubro Red
**T15**	Farm T	4+	8	Ubro Red
**T16**	Farm T	3	5	Ubro Red
**T17**	Farm T	1	3	Ubro Red
**T18**	Farm T	2	4	Ubro Red
**T19**	Farm T	2	4	Ubro Red
**T1**	Farm T	4+	7	Ubro Red

**T20**	Farm T	2	4	Ubro Red
**T2**	Farm T	4+	6	Ubro Red
**T4**	Farm T	1	3	Ubro Red
**T5**	Farm T	2	4	Ubro Red
**T6**	Farm T	0	2	None
**T7**	Farm T	4+	9	Ubro Red
**T8**	Farm T	3	5	Ubro Red
**T9**	Farm T	0	2	None
